# “Sometimes We Can’t Afford the Healthy Stuff”: Perceptions of Cardiovascular Disease Risk and Healthy Food Accessibility Among Black Women Living in Public Housing

**DOI:** 10.3390/ijerph22020252

**Published:** 2025-02-11

**Authors:** Alisia Sullivan, India M. Smith, Chanel D. Blue, Brandi M. White

**Affiliations:** College of Health Sciences, University of Kentucky, Lexington, KY 40506, USA; actayl2@uky.edu (A.S.);

**Keywords:** cardiovascular disease, food insecurity, public housing, African American women

## Abstract

African American women living in public housing carry a heavy burden of cardiovascular disease. Eating a heart-healthy diet is crucial to achieving optimal heart health, yet this health disparity population encounters major barriers to healthy eating. This study explored their perceptions of healthy eating and cardiovascular disease. Participants were recruited from public housing in a mid-sized city. Six 2-h focus groups with 32 women were conducted. Focus groups were analyzed using deductive coding. The major focus group findings focused on a limited access to affordable healthy foods. Participants also discussed the use of cost control measures to maximize household food budgets to access healthy foods and the ability to eat healthily. Our findings indicate that food insecurity persists for the populations most at-risk for cardiovascular disease. Plant-based diets may offer a culturally sensitive, innovative, and sustainable approach to reducing heart health risks, alleviating food insecurity, and promoting optimal health outcomes.

## 1. Introduction

Cardiovascular disease (coronary heart disease, heart failure, stroke, and hypertension) affects nearly half of all American adults ≥20 years of age (48.6%) [1]. Despite efforts to reduce cardiovascular disease rates in the United States, non-Hispanic African American (or Black American) women continue to have higher rates compared to their non-Hispanic White, Hispanic, and Asian counterparts (58.8%, 42.1%, 42,7%, and 42.5%, respectively) [1]. Low-income African American women, including those living in public housing, are at an even higher risk for cardiovascular disease [2,3]. Compared to the general public, public housing residents carry the burden of hypertension (37.9% and 26.5%, respectively), heart disease (19.8% and 12.8%), and obesity (41.01% and 23.2%) [4]. The reasons for these health inequities are complex and multifaceted, ranging from inadequately built and social environments [5,6,7,8], psychosocial stress and discrimination [9,10,11,12], and financial insecurity [13,14]. African American women in public housing are particularly vulnerable because, as a subgroup, they are less likely to be aware of cardiovascular disease risk factors [15].

The majority of risk factors for cardiovascular disease are modifiable through behavioral lifestyle interventions [16]. In addition to physical activity and abstaining from cigarette smoking, having a heart-healthy diet is crucial for the primary prevention and/or management of cardiovascular disease, especially hypertension [17,18]. A plant-based diet consisting of a higher intake of fruits and vegetables has been shown to reduce diabetes risk [19], lower blood pressure and hypertension risk [20], and potentially reverse coronary artery disease [21].

For health disparity populations such as low-income African American women, the ability to have a heart-healthy or plant-based diet is challenging because of limited socioeconomic resources. Because of the importance of diet and nutrition in reducing cardiovascular disease risk, it is crucial to understand, from the perspective of low-income African American women, their ability to access healthy foods. This information is important to understand to develop targeted public health and clinical interventions to promote cardiovascular health equity for this vulnerable population.

The United States Department of Agriculture (USDA) defines food insecurity as “the limited or uncertain availability of nutritionally adequate and safe foods, or limited or uncertain ability to acquire acceptable foods in socially acceptable ways” [22]. The national estimates of food insecurity in 2023 were 13.5% of households, including 5.1% having very low food security [23]. Estimates were higher for households with an income below the federal poverty line, with 38.7% households being food insecure [23]. The prevalence of food insecurity for residents of public housing nationwide is unknown. However, cross-sectional studies conducted with this population have provided insight, i.e., in the Boston Public Housing authority, food insecurity was found to be at 38.9% [24].

Food insecurity is a major risk factor for cardiovascular disease [25,26], and a disproportionate amount of low-income African American women experience food insecurity [13,14]. To help with the development of targeted cardiovascular risk reduction efforts, this study explored the perceptions of cardiovascular disease risk and food insecurity from the perspective of one subgroup of low-income African American women: those living in public housing.

## 2. Materials and Methods

A sample of 32 participants were recruited from majority African American-occupied public housing developments in central Kentucky. The university’s ethical review board approved this study (#58765).

In order to meet the eligibility criteria, participants had to be 18 years or older, self-identify as a non-Hispanic Black or African American woman, speak and understand English, and currently reside in public housing. All participants met the inclusion criteria and provided their consent to participate in this focus group study. Participant recruitment efforts included word-of-mouth, along with flyers posted in the local public housing development’s main offices. Participants who were interested in the study and felt that they would be eligible contacted study staff via a provided phone number. To ensure that all aspects of the inclusion criteria were met, participants completed an eligibility survey with study staff. Eligible participants were then invited to begin the study and join a pre-scheduled focus group.

Six total focus groups were conducted and led by the principal investigator (White), an African American woman, between May and June of 2019. For the discussion, a semi-structured focus group guide was used to help facilitate conversation (Table 1). Discussions took place during the evening time in a location that was convenient for the community. Focus groups lasted between 30 min and 1 h and 30 min in duration. Heart-healthy meals were prepared for participants in each focus group. Prior to starting the focus group discussions, participants completed a sociodemographic and health survey with questions from the 2018 Behavioral Risk Factor Surveillance System (BRFSS) for descriptive purposes. All focus groups were audiotaped. To thank participants for their time, they were provided with a USD 30 gift card at the focus group completion.

SAS 9.4 was used to generate descriptive statistics from the sociodemographic and health survey (SAS Institute, Inc. Cary, NC, USA). When describing continuous variables, means were used, and to describe categorical variables, frequencies were used. Research assistants transcribed the audiotaped focus groups verbatim and removed identifying information. Transcripts were analyzed using inductive content analysis by the study team (authors AS, IMS, CDB, BMW). Each team member independently coded data line-by-line to tag text segments and developed initial themes related to food access. The study team met to discuss initial themes and used group consensus to determine final themes. Team members then independently selected participants’ quotes and grouped them by theme. The study team met again to review compiled quotes and associated themes, and used group consensus to identify quotes to be included in final analysis.

## 3. Results

### 3.1. Participant Characteristics

Socio-demographic and health-related characteristics of study participants are summarized in Table 2. The mean age of participants was 49.9 years with a range from 32 to 64 years of age (standard deviation ± 10). Most of the participants had a high school education or higher (80%). A little over half of the participants were employed (61%) and earned less than USD 800 per month (60%). High blood pressure (41%) and hypertension (31%) were the most reported CVD-related risk factors and conditions, followed by high cholesterol (28%) and type 2 diabetes (22%). Almost a third of participants were current smokers (28%).

### 3.2. Focus Group Findings

Three major themes related to food insecurity and cardiovascular disease risk were identified: (1) lack of access to affordable healthy foods; (2) use of cost control measures to maximize household food budgets to access healthy foods; (3) and self-efficacy in healthy eating behaviors.

#### 3.2.1. Lack of Access to Affordable Healthy Foods

The lack of affordable healthy foods was a major discussion topic when discussing risks to cardiovascular disease. Participants in the study perceived that the higher costs associated with healthier food items made the purchase of those items outside of their financial wherewithal. A participant summarized this perception when stating: “To me it’s all finances to eat healthy”. Participants acknowledged that their limited household budgets also influenced the quality of food that was purchased. Participants asserted that nutrition assistance programs such as the Supplemental Nutrition Assistance Program (SNAP) were not sufficient to overcome the food insecurity that was experienced by members within their household. One participant stated:

“Eating healthy is affordability. A lot of people, when you talk to them, like, I can’t afford to buy this organic stuff. You know? Everything processed is easier, even with food stamps. Now, you can buy the processed chicken already fried in the little containers, approved by the government. The government’s approving us to eat this”.

When shopping, another participant stated:

“Well I don’t never go down the candy bar aisle, I don’t go down the soda pop aisle, I don’t go down the chip aisle, but when I have my grandkids and I take them with me, them the aisles that I go down. But everything on the sideline of the wall is all the healthy stuff when you go in the store. It ain’t the stuff in the middle, it’s the stuff in the sideline. Even though it’s gonna cost more, that’s where it’s at”.

Several other participants supported this statement by affirming that limited economic resources and limited access to healthy foods may be why African American women have more cardiovascular disease risk factors compared to other groups of women. For example, one participant stated, “Our income is very limited… I love fresh vegetables, but sometimes I have to get them frozen. I do not get canned, canned is the least that I do”. One participant bleakly stated: “[African American women have more heart disease because of] financial reasons, not having enough money to buy the proper diets”. Another participant echoed this belief: “Sometimes we can’t afford the healthy stuff”.

Other participants discussed competing financial priorities as a contributing factor to the lack of access to affordable healthy foods. For example, one participant stated:

“The cash, it’s like, because if your kids need something, you have to buy it for them. Then you have to buy something for the home and for yourself too, so money is everything and you got food stamps, but you want to buy clothes. Your food stamps cannot buy it, you have to [use] cash. There’s a lot of different stuff, even if you do have food stamps but there’s nothing on the food card, then you want to go to get something, then you have to get cash to pay for what you have to buy, because you have to wait for the next month. And sometimes not everybody got food stamps”.

#### 3.2.2. Use of Cost Control Measures

The use of cost control measures to maximize household food budgets was a common strategy to increase access to healthy foods. Participants described the utilization of several different cost savings measures. For example, one participant stated:

“What I do is I look every week [for coupons]… So, I’ll buy a lot of fish. They [her children] love baked chicken, salads. Last time, we had spaghetti, breadsticks, and tea, but what I did was, I used gluten free noodles, put carrots in, gave them a nice serving, and that was it”.

The use of coupons enabled her to afford healthier food items, which she then incorporated into her families’ meals.

Another participant asserted that she also maintained a food budget and engaged in comparison shopping:

“I like to cook. I cook all the time. We only go out every once in a while because sometimes I don’t wanna cook. I do budget. I feel like budgeting is important and catching sales. I get stuff from Save-A-Lot, but I also get stuff from Kroger, and Walmart, but I get it when it’s on sale. If I can get the good stuff for cheaper, I’m going to do that”.

By maintaining a food budget, shopping at discount grocery store chains, and engaging in comparison shopping, this participant was able to maximize her food budget and increase her family’s access to healthy foods.

Another participant stated, “If I can get the name brand stuff for cheaper, I’ma go for the name brand. Some stuff you can have the cheap and it’s better than the expensive and sometimes the expensive is better than the cheap, depending on what it is”.

This participant compared different food brands for quality and price to select healthier options that fit within her food budget.

Another participant engaged in food portioning to increase her access to healthier food options:

“I’ma tell y’all, I was at Kroger store the other day. I got some cherries, I got some grapes, and I went in the line and paid for them, and I budget. I said oh, this just me, take some off there, and put it back. Because I don’t have to buy the whole bag, you can buy what you want. So, I plucked it off; it’s expensive, and I got what I wanted, to make what that price is I wanted to pay”.

By removing the grapes and/or cherries from the pre-packaged bags, the participant was able to reduce the total weight and cost of her purchase to purchase only the amount she needed and manage her food budget.

#### 3.2.3. Self-Efficacy in Healthy Eating Behaviors

Several participants voiced that they believed they could access healthy foods if they wanted to. For example, one participant stated, “Yeah, we have access to it [healthy foods], but then you have to go to Kroger and pick healthy foods out. It’s there we just have to choose it. Stay out the center aisle [location of cheaper, processed foods]”.

Participants agreed that suboptimal consumer choices contributed to unhealthy eating habits and that conscious decisions needed to be made to purchase healthier food items to improve dietary behaviors.

Another participant acknowledged the role of individual decision making in purchasing healthier food items; however, she also mentioned the lack of appealing healthy food options:

“You can eat the same thing; it doesn’t have to be in a tray. We have access to the farmers market… You can buy it individually. It may not be what you want at that time. I think we do have access to healthy food, it may just not be what we want. Those of us that have families, you have to buy what you can afford, that’s going to stretch, to afford to feed your whole family. They got access to it, but you have to make that last. You have to make it stretch because all kids ain’t goin’t eat it. If you don’t eat that, your kid don’t eat that. Kids can only do what their parents allow them to do. You gotta go to household first, that’s where the problem is”.

This participant’s difficulty in accessing appealing, healthy food options that align with her family’s preferences impacts her self-efficacy regarding healthy eating. To minimize food waste, due to her family’s reluctance to eat certain foods, she often opts for more familiar, less healthy choices to maximize her limited food resources.

## 4. Discussion

Our study participants identified major barriers to healthy eating to reduce cardiovascular disease risk that revolved around food insecurity. A lack of access to affordable, healthy, quality foods was a common theme, and most participants perceived healthy foods as more costly. Federal programs to reduce food insecurity, such as SNAP, were perceived as being ineffective in increasing their access to healthy foods. The inaccessibility of healthy foods was also perceived as a contributing factor to African American women having higher rates of cardiovascular disease compared to other racial/ethnic groups. Participants combatted food insecurity by using cost control measures to increase their budgets to access healthy foods and many believed that they had the ability to eat healthy.

Similar studies examining food insecurity among low-income African Americans found high levels of reported major barriers to accessing affordable healthy foods [14,27]. One study was a cross-sectional examination of associations between food insecurity, body weight, psychosocial factors, and eating behaviors among low-income African American families [14]. They found that participants that perceived healthy foods as being expensive were more likely to be food insecure. Similarly, our participants who reported barriers to accessing healthy foods also reported that healthy foods were more costly. Over one-third of participants in their study were food insecure (41.6%) [14]. Food insecurity was significantly associated with unemployment or underemployment (part-time work) and the belief that healthy foods were not accessible.

Wilcox et al. also found high rates of food insecurity in a sample of low-income African Americans [27]. Food insecurity was significantly associated with having a poor diet quality. More specifically, only ~17% of food insecure participants met the daily dietary guidelines for both daily fruit and vegetable consumption. Not surprisingly, because the sample was economically disadvantaged, ~16% food secure participants also reported low rates of fruits and vegetables [27]. Participants in our study reported economic barriers to eating fruits and vegetables. They also perceived this disparity as a major contributing factor to their increased risk for cardiovascular disease.

Interventions (e.g., SNAP) designed to provide more economic resources to buy sufficient food and reduce food insecurity were seen as a failure by our study participants. As one of our participants stated, participation in such programs does not improve diet quality to prevent and mitigate chronic conditions such as cardiovascular disease. National estimates of eating patterns indicate that SNAP participants have poorer diets compared to nonparticipants, including a lower consumption of fruit and vegetables [28]. This diet quality disparity consequently increases their risk for cardiovascular disease and other metabolic conditions. A study comparing heart-healthy diets by SNAP status found that SNAP participants had the worst heart-healthy diet score compared to income-eligible participants and higher-income participants [29]. This further highlights the diet disparities experienced by SNAP participants.

Participants reported operating within limited financial means, often purchasing off-brand food items to maximize their purchasing power, shopping at discount grocery stores, engaging in comparison shopping, and employing budgeting strategies. Despite utilizing food subsidy programs (e.g., SNAP) and financial strategies to increase food affordability, the costs of healthy foods were prohibitive, and a barrier to adopting a healthier diet. Strategies aimed at improving dietary choices among African American women in public housing must be multifaceted and address health behaviors, diet, and food affordability. A promising approach to alleviate the burden of food affordability and improve the dietary consumption of healthy foods may be found in plant-based diets. Plant-based diets are defined as “dietary patterns that emphasize foods of plant origin, rather than meats and animal byproducts” [30]. Examples of plant-based foods include whole foods like fruits, vegetables, nuts, whole grains, and legumes. The extant literature has found that, among African Americans, leafy green vegetables (e.g., collard greens, spinach, kale, mustard greens, turnip greens) are the most often consumed [31,32,33,34,35]. Integrating culturally familiar plant-based foods into the diets of AA women can encourage the adoption of healthier eating habits. Emphasizing familiar foods can make the transition to a plant-based diet more accessible and appealing.

A review conducted by Sterling and Bowen also found that plant-based diets adherence among African Americans reduced risk factors associated with heart disease [30]. Plant-based diets were also found to be more cost-effective compared to other diets (e.g., standard, Mediterranean) [36,37]. In a study conducted by Kahleova et al., adopting a vegan diet (e.g., fruits, vegetables, grains, and legumes) compared to a Mediterranean diet resulted in a 19% reduction in food costs [38]. To further support the adoption of healthier dietary behaviors, interventions should focus on enhancing self-efficacy and positive attitudes toward purchasing and consuming plant-based foods [39]. When exploring the dieting behaviors among low to moderately plant-based African American women, attitude and self-efficacy toward purchasing deep green leafy vegetables were strongly associated with the intention of African American women to purchase dark green leafy foods [40]. Diets that consist of more cost-effective food options may be more appealing, and empower African American women in public housing to make healthier dietary choices, decreasing their risks for heart disease.

Our findings also reveal the importance of intervening at both the individual/household level, as well as the community and policy levels to promote food security for optimal heart health. For African American women living in public housing, this involves working with them and key stakeholders, including public health agencies, community-based clinics, public health departments, and community leaders to develop and evaluate sustainable solutions that provide access to healthy foods. Planning for such solutions requires an “outside-of-the-box” perspective that builds on existing community assets to create *food paradises* rather than *food deserts*. Our anecdotal work with our public housing communities shows promise with the new generation of African American farmers who are social justice warriors and often eager to work on efforts to increase access to healthy foods for minoritized communities. Community gardens are promising interventions that have the potential to reduce cardiovascular disease risk and eliminate food insecurity; however, food safety training is important for proper management [41].

In addition, knowing the location of the nearest grocery store or farmer’s market alone is not enough to ensure food access. Increasing health literacy and financial resources are essential to eliminate food insecurity. Health literacy should involve teaching individuals how to shop for foods, prepare and cook healthy meals, and store fresh fruits and vegetables. For SNAP participants, the Expanded Food and Nutrition Education Program and the Supplemental Nutrition Assistance Program (SNAP-Ed), which target low-income families and individuals, are pathways to increase health literacy. For African Americans, this knowledge must incorporate culturally relevant and customary foods to increase adherence to a heart-healthy diet [42,43,44]. Financial resources for individuals to buy healthy foods is also important to eliminate food insecurity. Existing food assistance programs such as SNAP must provide additional discounts or funds for participants to buy healthy foods. Encouraging buying fresh foods through farmers markets and community-supported agriculture, and subsidies for healthier food options, are potential ways to lower the cost of healthy foods.

To advocate for health equity, it is important to note that African American women have been historically disenfranchised in the United States. Stereotypical depictions of Black women as “lazy welfare queens” has been used to perpetuate federal policies and programming that seeks to reinforce the ideology that Black women are ineffective at managing their households [45,46]. When faced with food insecurity, oftentimes individuals are challenged to “make it work”. We place the onus on those who are impacted to do a better job of managing their limited resources. We fail to acknowledge the reality of their lived experience, i.e., housing located in food deserts, limited access to grocery stores due to lack of viable transportation, limited financial resources. Recognizing our personal biases surrounding race, gender, and socioeconomic status is needed to promote health equity.

### Study Limitations

This study is not without limitations. Our findings may not be generalizable to African American women living in public housing in other communities. While inclusion criteria included self-identifying as an African American or Black American women, some of the participants may have not been generationally rooted in the United States (that is, their ancestors suffered under US chattel slavery). We also did not quantitatively assess food security for participants. Despite these study limitations, this study provides new information about the pre-contemplative mindset of African American women living in public housing, as it pertains to perceptions of their diet and cardiovascular disease.

## 5. Conclusions

In our sample of African American women living in public housing, participants had difficulty accessing affordable healthy foods when discussing cardiovascular disease risk factors despite being enrolled in food assistance programs. These findings demonstrate the need to develop targeted interventions and policies that increase access to healthy foods in economically disadvantaged communities and increase knowledge to optimize heart health.

## Figures and Tables

**Table 1:** Semi-structured interview guide used during focus group discussion.

No.	Question and Probes
1	When you hear the words heart disease, what comes to mind?
2	As a Black woman, what do you think your chances are of getting heart disease or stroke?
3	What can you do to prevent your risk for heart disease or stroke?
4	Tell me about some barriers to preventing heart disease and stroke.
5	Describe to me some of the things that KEEP you from preventing heart disease and stroke.
6	Tell me about some of your strengths that help you prevent heart disease and stroke.
7	Tell me more about strengths that could be useful to prevent heart disease and stroke.
8	How would you prefer to have information provided to you about heart disease and stroke?
9	What heart disease and stroke information do you want to learn about?
10	If a program to prevent heart disease and stroke was developed specifically for African American women living in public housing, what would it look like?

**Table 2:** Socio-demographic and cardiovascular-related risk factors and health behavior characteristics of study participants (N = 32).

Characteristic	Percent (%)
Sociodemographic Characteristic	
Marital status: single or never married	50
Educational attainment: high school or higher	80
Employment status: employed	61
Monthly income: less than USD 800	60
Cardiovascular-related risk factors and conditions	
High blood pressure (not diagnosed hypertension)	41
Hypertension	31
Current smoker	28
High cholesterol	28
High blood sugar (diabetes)	22
Heart problems	19

## Data Availability

The data presented in this study are not available due to privacy concerns.
